# AhR and STAT3: A Dangerous Duo in Chemical Carcinogenesis

**DOI:** 10.3390/ijms26062744

**Published:** 2025-03-18

**Authors:** Marco Minacori, Sara Fiorini, Monia Perugini, Annamaria Iannetta, Giorgia Meschiari, Silvia Chichiarelli, Fabio Altieri, Pier Giorgio Natali, Margherita Eufemi

**Affiliations:** 1 Department of Biochemical Science “A. Rossi Fanelli”, Faculty of Pharmacy and Medicine, Sapienza University of Rome, P.le Aldo Moro 5, 00185 Rome, Italy; mminacori@unite.it (M.M.); sara.fiorini@uniroma1.it (S.F.); giorgia.meschiari@uniroma1.it (G.M.); silvia.chichiarelli@uniroma1.it (S.C.); fabio.altieri@uniroma1.it (F.A.); margherita.eufemi@uniroma1.it (M.E.); 2 Department of Bioscience and Agro-Food and Environmental Technology, University of Teramo, Campus “Aurelio Saliceti”, Via R. Balzarini 1, 64100 Teramo, Italy; mperugini@unite.it (M.P.); aiannetta@unite.it (A.I.); 3 Collegium Ramazzini, Castello di Bentivoglio, Via Saliceto, 3, 40010 Bologna, Italy

**Keywords:** AhR, STAT3, chemical carcinogenesis

## Abstract

Human chemical carcinogenesis is a multistage process where chemicals or their metabolites cause irreversible changes in normal cell physiology, eventually leading to uncontrolled proliferation, transforming a normal cell into a cancerous one. Signal transducer and activator of transcription 3 (STAT3) is a cytoplasmic transcription factor that regulates cell proliferation, differentiation, apoptosis, angiogenesis, inflammation, and immune responses. Its aberrant activation triggers tumor progression by promoting the expression of oncogenic genes; thus, STAT3 is classified as an oncoprotein. The aryl hydrocarbon receptor (AhR) is a ligand-activated transcription factor that responds to a wide variety of chemicals, including carcinogens like dioxins, inducing genes associated with detoxification, proliferation, and immune regulation. Recent reports show that AhR plays a critical role in cancer development and maintenance. AhR may interact with signaling pathways, like the STAT3 pathway, which mediates the carcinogenic effects of several pollutants. Various chemical agents, such as industrial waste and hydrocarbon compounds, can alter the expression or signaling activity of AhR and STAT3 pathways, leading to different types of cancers. Understanding the complex STAT3-AhR network in the regulation of chemical carcinogenesis could open new avenues for cancer prevention or treatment, particularly in personalized medicine, aiming to improve life expectancy and achieving a complete cure.

## 1. Chemical Carcinogenesis

**Figure 1:**
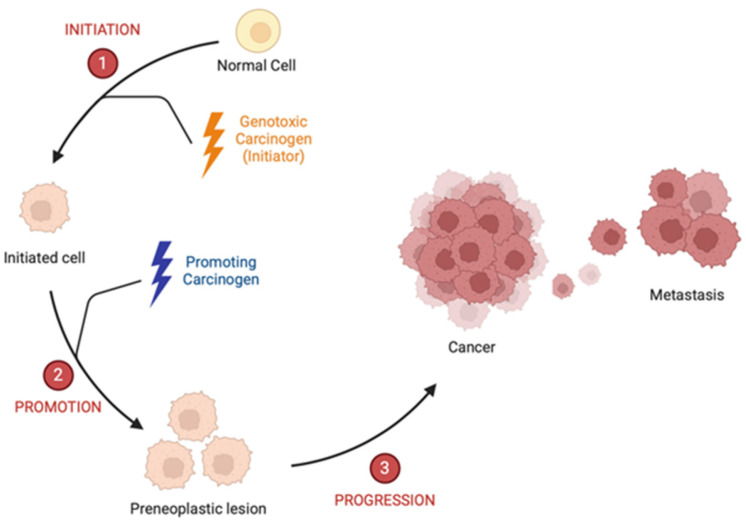
Schematic representation of three phases carcinogenesis.

Cancer remains one of the leading causes of mortality worldwide, with over 19 million new diagnoses and approximately 10 million deaths annually, accounting for one in six deaths globally [1]. The risk of carcinogenesis is significantly influenced by exposure to certain environmental chemicals [2]. Chemical carcinogenesis refers to the series of cellular events induced by chemical agents or their metabolites, which result in irreversible alterations to normal cellular physiology, ultimately leading to the transformation of a normal cell into a malignant one [3]. Human exposure to hazardous chemical substances can modulate a variety of biological and molecular processes, including gene expression, DNA repair mechanisms, hormone synthesis and function, and inflammation [4]. Agents capable of causing DNA damage, and consequently alterations in gene expression, are classified as genotoxic substances [5]. In contrast, non-genotoxic compounds with estrogen-like and mitogenic properties, known as xenoestrogens, function as endocrine disruptors, leading to widespread dysregulation of cellular signaling pathways [6]. Both categories of molecules are implicated in the induction of carcinogenesis. These modifications, if not resolved, have detrimental effects, as they will drive the three stages of the chemical carcinogenesis process: initiation, promotion, and progression [7,8]. The initiation phase is rapid and generally irreversible. Evidence indicates that initiation typically results from one or more mutations in cellular DNA, often induced by chemical agents through covalent interactions between electrophilic derivatives of carcinogens and DNA. The promotion phase is reversible and does not involve direct DNA damage. Instead, it is characterized by the modulation of gene expression, which leads to an increased cell population through enhanced cell division and/or the inhibition of apoptotic cell death. As cellular proliferation continues, additional mutations may accumulate in preneoplastic cells, ultimately driving the progression toward neoplasia. Therefore, carcinogens possess both initiating and promoting activities [9]. The third phase, progression, involves further genomic alterations and, unlike the promotion phase, is irreversible. Progression marks the final stage of neoplastic transformation, during which both genetic and phenotypic changes occur, accompanied by an increase in cell proliferation [10]. This results in the rapid growth of the tumor, with cells acquiring additional mutations that confer invasive and metastatic potential. The three stages of chemical carcinogenesis schematized in Figure 1 are characterized by specific cellular activities induced by the activation or alteration of certain signaling pathways [11].

In the case of chemical carcinogenesis, crucial roles are played by the pathways triggered by the AhR (Aryl Hydrocarbon Receptor), responsible for inducing xenobiotic detoxification processes [12], and by those of the STAT3 (Signaling Transductor and Activator of Transcription), an oncoprotein. The pathways of STAT3 are not only constitutively activated in most cancers but also play a crucial role in cellular responses to substances such as organochlorine pesticides [13,14,15]. Therefore, in order to gain a better understanding of the mechanisms regulating chemical carcinogenesis, it is necessary to study and clarify the pathways mediated by AhR and STAT3, as well as their potential interplays.

## 2. Aryl Hydrocarbon Receptor (AhR)

Since the discovery of the AhR in the 1970s, its study has been at the forefront of toxicology, as it mediates the effects of many environmental pollutants, particularly halogenated aromatic hydrocarbons. Initial research on AhR focused on understanding its canonical signaling pathway, triggered by its prototypical ligand, 2,3,7,8-tetrachlorodibenzo-p-dioxin (TCDD) [16]. Subsequently, it was observed that AhR is involved in various physiological processes such as immune system regulation, hepatic homeostasis, cardiac development, wound healing, cell proliferation and apoptosis, metabolic diseases, and tumor promotion. Further studies aimed at elucidating these additional activities of AhR demonstrated that it also mediates other signaling pathways, one of which is defined as non-canonical. Together with the canonical pathway, they represent the genomic pathways of AhR, as their ultimate targets are genes located in the nuclear compartment, while the non-canonical pathway operates exclusively in the cytosol [17].

### 2.1. AhR Genomic Canonical Pathway

Exposure to xenobiotic substances, whether of natural origin, such as polyphenols, or synthetic, such as halogenated aromatic hydrocarbons (HAHs) and polycyclic aromatic hydrocarbons (PAHs), would be incompatible with life if organisms did not possess a detoxification system comprising proteins capable of inducing the metabolic processes necessary for the processing of these substances. A key player in this context is the aryl hydrocarbon receptor (AhR), whose specific substrates are xenobiotic substances with a planar aromatic structure, to which the aforementioned classes of compounds belong [18]. The AhR is traditionally defined as a ligand-dependent transcription factor involved in the biotransformation and carcinogenic effects of environmental toxins, such as 2,3,7,8-Tetrachlorodibenzo-p-dioxin (TCDD) and polycyclic aromatic hydrocarbons (PAHs) [19].

The AhR was first identified in the mid-1970s as a cytoplasmic receptor that binds 2,3,7,8-tetrachlorodibenzo-p-dioxin (TCDD) with extremely high affinity [18]. It was subsequently shown that PAHs also bind to AhR, inducing the expression of metabolizing enzymes, including cytochrome P450 enzymes, particularly members of the CYP1A1, CYP1B1, and CYP2A1 families [20]. Upon ligand binding, AhR translocates into the nucleus, where it regulates the transcription of genes involved in the metabolism of xenobiotic substances, such as environmental pollutants, pharmaceuticals, and others. Therefore, AhR functions as a cytosolic receptor for low molecular weight chemical molecules.

AhR is expressed in all organs and tissues but is particularly abundant in those most exposed to chemical substances, such as the liver, lungs, skin, gastrointestinal tract, and placenta [21]. The canonical pathway of AhR begins when the receptor is in a latent state in the cytosol, as part of a multiprotein complex composed of chaperone proteins, such as heat shock protein 90 (Hsp90), co-chaperone HSP23, prostaglandin E synthase 3 (PTGES3, also known as p23), and X-associated protein 2 (XAP2) from the hepatitis B virus. These proteins are essential for the proper folding of AhR, ensuring specific ligand recognition and facilitating its transcriptional activity [22].

Ligand binding induces a conformational change in AhR, exposing its nuclear localization sequence (NLS), which allows its translocation into the nucleus in association with importins. In the nucleus, AhR replaces its interaction with Hsp90 by binding to aryl hydrocarbon receptor nuclear translocator (ARNT), also known as HIF-1β. The AhR/ARNT complex binds to xenobiotic response elements (XREs) in promoter sequences, initiating the transcription of target genes [23].

The proper physiological function of the canonical AhR pathway is regulated through an on–off control mechanism, governed by two feedback systems. The first feedback system involves the release of AhR back into the cytosol after it fulfills its function as a transducer and transcription factor, followed by its degradation via the 26S proteasome pathway [24]. The second feedback system is mediated by AhR binding to XREs, which induces the transcription of the AHRR gene, encoding the aryl hydrocarbon receptor repressor (AHRR). AHRR regulates AhR activity through a negative feedback loop. Specifically, in the nucleus, AHRR dimerizes with ARNT, leading to dissociation or preventing its association with AhR, thereby inhibiting AhR’s function as a transcription factor [25].

The aryl hydrocarbon receptor is an indispensable protein, essential for survival in the face of environmental insults caused by xenobiotics. Phylogenetic studies support this function, indicating that AhR is an ancient protein with a common ancestor across species dating back approximately 600 million years [26].

### 2.2. AhR Genomic Non-Canonical Pathway

The non-canonical AhR pathway was discovered during a DNA microarray study aimed at identifying its target genes. The results from this study revealed that some genes responsive to AhR activation do not contain canonical xenobiotic response element (XRE) sequences. One such example is the gene encoding plasminogen activator inhibitor-1 (PAI-1), where TCDD-induced transcriptional activation is attributed to a regulatory region lacking a canonical XRE. Recently, a novel non-consensus XRE (NC-XRE), containing a 50-GGGA-30 tetranucleotide motif, has been characterized within the PAI-1 promoter [27]. This sequence explains the binding of AhR to DNA independently of the aryl hydrocarbon receptor nuclear translocator (ARNT) protein. In these studies, it was confirmed that AhR binding to the NC-XRE occurs only after AhR has dimerized by interacting with a newly identified protein, Kruppel-like factor 6 (KLF6), which serves as a novel AhR partner. KLF6 (also known as Zf9 or CPBP) is a ubiquitously expressed Cys2-His2 transcription factor belonging to the Kruppel-like family of zinc finger transcription factors, which regulate processes such as cell proliferation, signal transduction, differentiation, and development [28]. The expression of p21Cip1, a cyclin-dependent kinase inhibitor in the G1-phase of the cell cycle, is regulated by the NC-XRE-mediated AhR-KLF6 complex. This accounts for the increased expression of p21Cip1 following environmental exposure to TCDD, with the AhR-KLF6 complex playing a critical role in controlling cell cycle processes and the deleterious effects of TCDD [29]. The canonical and non-canonical AhR pathways are both considered genomic pathways, as upon ligand interaction, AhR translocates to the nucleus to initiate specific transcriptional processes.

### 2.3. AhR Non-Genomic Pathways

In contrast, in the non-genomic pathway, AhR typically remains cytosolic. For example, following TCDD binding, AhR induces calcium release from the endoplasmic reticulum and facilitates extracellular calcium uptake, leading to an increase in cytosolic calcium concentrations. This, in turn, activates protein kinase C (PKCα) [30]. Alternatively, AhR can activate the tyrosine kinase Src, one of the components of the AhR complex in its latent state which is released following the AhR–ligand interaction, subsequently leading to its activation. Src kinase activity leads to the rapid activation of MAP kinases (ERK1 and ERK2) and focal adhesion kinase, resulting in modifications to the cell’s adhesion properties through the disruption of focal adhesion points [31].

These non-genomic activities converge to regulate various pathophysiological processes, including cell proliferation, apoptosis, differentiation, adhesion, migration, pluripotency, stemness, and inflammation. Additionally, AhR interacts with several signaling pathways, including EGFR, JAK/STAT3, HIF-1α, NRF2, NF-κB, estrogen receptor (ER), and androgen receptor (AR). The interaction between AhR and these signaling pathways occurs through several mechanisms, such as activation of different signaling proteins by Src family kinases, transcriptional regulation of gene expression, and direct protein–protein interactions among key signaling molecules within these pathways. In turn, signaling pathways can modulate the expression or activation of AhR, creating a feedback loop that influences signal intensity within tissues [32].

Alterations in the expression or activity of AhR can significantly influence the signaling activities of these various pathways, thereby promoting carcinogenesis. Various chemical agents, including tobacco, industrial chemical waste, hydrocarbon compounds, pharmaceuticals, as well as physical and biological agents, can modify AhR expression or its signaling activity, activating AhR-mediated carcinogenesis [33]. This process is implicated in the development of several types of cancer, including lung, breast, colorectal, liver, prostate cancer, and leukemia [34]. Understanding the mechanisms by which these factors regulate the AhR-mediated carcinogenic process may assist scientists and clinicians in developing personalized therapies aimed at slowing cancer progression and improving patient survival outcomes.

## 3. Signal Transducer and Activator of Transcription 3 (STAT3)

As extensively described in the literature for its involvement in tumor processes, STAT3 is a multifaceted protein capable of orchestrating a wide range of cellular activities, including proliferation, angiogenesis, growth, apoptosis, epithelial-mesenchymal transition (EMT), energy metabolism, and mitochondrial activity through its canonical and non-canonical pathways [35].

### 3.1. STAT3 Canonical Pathway

Following the activation of the cytokine receptors, growth factor receptor and cytosolic kinase, STAT3 is phosphorylated at Tyrosine 705 (Y705). This post-translational modification promotes STAT3 dimerization, which subsequently triggers its translocation to the nucleus, where it activates a gene expression program specific to each signaling pathway it mediates. The cellular responses to STAT3 activation are several, encompassing processes such as proliferation, differentiation, angiogenesis, apoptosis, inflammation, and immunosuppression [36].

Under normal physiological conditions, STAT3 activation is tightly regulated, being transient and strictly controlled through inhibitory mechanisms involving proteins such as SOCS3 (Suppressor Of Cytokine Signaling 3) and PIAS3 (Protein Inhibitor of Activated STAT3), as well as phosphatases like SHP1 and SHP2 [37]. However, when the regulation of STAT3 is not carefully controlled, constitutive signaling of this protein may dominate, causing it to function as an oncoprotein and playing a central role in carcinogenesis. Literature suggests that STAT3 is involved in all three phases of chemical carcinogenesis. Specifically, constitutive phosphorylation of STAT3 at Tyr705 has been observed in a broad spectrum of cancers, including prostate, breast, gastric, bladder, lung, intestinal, and ovarian cancers, as well as in lymphoma and glioma [38,39].

The various terms used to describe STAT3, such as pleiotropic, multifaceted, and hub, highlight its involvement in numerous cellular activities and its distribution across multiple cellular compartments, depending on the role it performs. STAT3 was first identified in 1994 as an acute phase response factor (APRF) involved in interleukin-6 (IL-6) signaling, acting as a mediator of the inflammatory response [40]. Subsequent studies revealed its pivotal role as a convergence point for multiple signaling pathways [41], as evidenced by its phosphorylation at Tyr705, dimerization, and nuclear translocation, thus defining the canonical STAT3 signaling pathway.

### 3.2. STAT3 Non-Canonical Pathways

Since 1994, additional cellular activities of the STAT3 protein have been identified, which are categorized as non-canonical pathways. Among the non-canonical pathways associated with carcinogenesis is the one involving unphosphorylated nuclear STAT3 (USTAT3). The function of USTAT3 is to regulate genes, such as RANTES, IL-6, IL-8, MET, and MRAS, by binding to unphosphorylated NFκB (U-NFκB), thereby competing with IκB. The resulting U-STAT3/U-NFκB complex accumulates in the nucleus with the assistance of STAT3′s nuclear localization signal, leading to the activation of a subset of NFκB-dependent genes, including those previously mentioned [42]. Moreover, USTAT3 has been shown to interact with Heterochromatin Protein 1 (HP1), stabilizing heterochromatin. This function contrasts with that of the phosphorylated Tyr705 (pY705) isoform, which, via the JAK receptor, increases the levels of phosphorylated STAT3 (pSTAT3) while decreasing USTAT3, thereby inducing heterochromatin instability—a hallmark of tumorigenesis. Literary evidence demonstrates that USTAT3 can translocate to the nucleus and predominantly reside there in various mammalian cells during the quiescent phase, when STAT proteins are not phosphorylated and there are no activation stimuli. This suggests that, under normal cellular homeostasis, USTAT3 may play a tumor-suppressive role by promoting heterochromatin stability through its interaction with HP1 [43].

Another non-canonical cellular function of STAT3 involves its metabolic role within the mitochondria. STAT3 has been shown to modulate the activity of mitochondrial complexes I, II, and V in the electron transport chain, thereby regulating cellular redox homeostasis and energy metabolism [44]. This function is linked to a specific post-translational modification, the phosphorylation at the Ser727 residue. While this phosphorylation is not essential for STAT3′s translocation into the mitochondria, it is critical for its mitochondrial functions and is associated with oxidative stress stimuli [45]. Within the mitochondria, STAT3 influences the electron transport chain (ETC) and the mitochondrial permeability transition pore (MPTP), thus affecting mitochondrial membrane potential (ΔΨ), proton gradient (ΔH+), ATP production, reactive oxygen species (ROS) levels, and cell death [46,47]. Some authors have also suggested that STAT3 influences mitochondrial energy metabolism through the canonical pathway, where its constitutive activation promotes aerobic glycolysis via induction of HIF-1α and PKM2, forming a feedback loop involving STAT3, PKM2, and HIF-1α [48].

In addition to its mitochondrial functions, STAT3 is implicated in several other non-canonical roles, such as the regulation of calcium release from the endoplasmic reticulum (ER). By interacting with the Ca^2+^ channel IP3R3, STAT3 modulates Ca^2+^ flux between the ER and mitochondria, thereby promoting apoptosis [49].

Other non-transcriptional and non-canonical roles of STAT3 include its interaction with Stathmin and its localization within lysosomes. In the first case, STAT3 mediates the stabilization dynamics of microtubules, which are essential components of cell architecture, contributing to processes such as proliferation, differentiation, and migration. This occurs through a functional interaction with Stathmin, a protein involved in microtubule destabilization [50]. Recently, STAT3’s role in lysosomes has also been described, both in modulating lysosome-mediated cell death and in its association with vacuolar H+-ATPase. Through this interaction, STAT3 regulates cytosolic and lysosomal pH by facilitating the flow of H+ from the cytosol to the lysosome, resulting in cytosolic alkalinization and lysosomal acidification [51]. Both of these activities are consistently correlated with a cancerous cellular state.

### 3.3. STAT3 Pollutants

Environmental pollutants, including organochlorine pesticides (OCPs), phthalates, per- and polyfluoroalkyl substances (PFAS), asbestos, heavy metals, and others, are chemical compounds released into the ecosystem that can induce a range of diseases in organisms exposed to them. In fact, within organisms, pollutants disrupt the physiological and pathological balance by bioaccumulating, promoting oxidative stress, inflammation, genomic alterations, mutations, epigenetic modifications, mitochondrial dysfunction, endocrine disruption, and alterations in cellular communication [52]. Many of the mechanisms induced by pollutants are mediated by the STAT3 protein. In fact, the STAT3 protein can be activated directly by environmental pollutants and through the activation of specific receptors such as Gp130/Jak2 [53], EGFRs [54] or indirectly by the ROS species [54]. Indeed, a search on PubMed for the term “STAT3 pollutant” reveals 235 results (February 2025), highlighting the significant role this protein plays in chemical carcinogenesis. Given that many cellular responses induced by chemical substances mirror those involved in carcinogenesis, a process in which STAT3 plays a pivotal role during all three phases (initiation, promotion, and progression) [55,56,57], it is reasonable to hypothesize the protein’s direct and indirect involvement in chemical carcinogenesis.

## 4. STAT3-AhR Interplays

**Figure 2:**
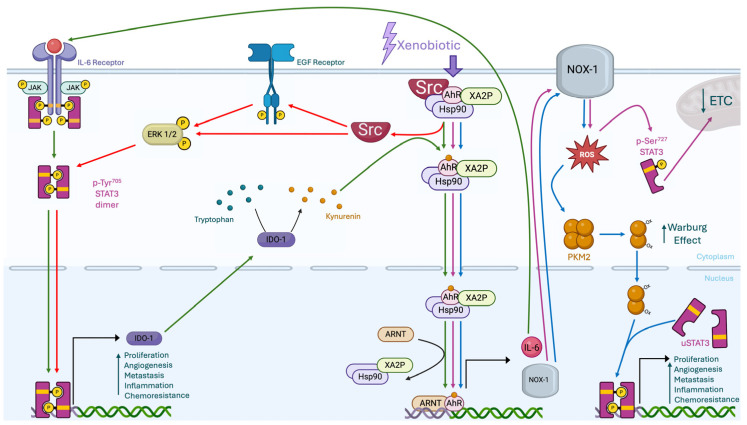
The panel schematically outlines the interplays between the AhR and STAT3 proteins. Each interplay follows a distinct color: the green one highlights the IDO-AhR-IL-6-STAT3 loop, the red one represents the AhR-Src-STAT3 interaction, the purple one depicts the AhR-NOX1-ROS-STAT3 axis, and the blue one illustrates the AhR-NOX1-ROS-PKM2-STAT3 pathway.

The interplay between proteins or cellular pathways represents, within the cell, a functional strategy to expand the activities of a single protein. This interplay creates a protein network from which the amplification of cellular responses to stimuli arises, making cellular adaptation to new conditions highly efficient. From Figure 2, for instance, which schematically illustrates the interplay between AhR and STAT3 proteins following the stimulation by a xenobiotic, it can be inferred how these networks amplify the cellular response in the process of chemical carcinogenesis.

### 4.1. Direct Interplay Between STAT3 and AhR

In addition to classic ligands, such as xenobiotic substances, it has been shown that endogenous ligands, including lipoxin 4A, a metabolite of arachidonic acid, biliverdin and bilirubin two heme metabolites and tryptophan metabolites such as FICZ (6-formylindolo [3,2-b]carbazole) and kynurenine, can also activate AhR [58]. The main pathway of tryptophan metabolism involves IDO1 (indoleamine-2,3-dioxygenase-1 or tryptophan-2,3-dioxygenase), which produces kynurenine, an agonist of AhR. IDO is an enzyme that is constitutively overexpressed in various tumors and plays a critical role in initiating the immunosuppressive activity of regulatory T cells (Tregs) [59]. IDO is regulated by the canonical activation of STAT3, specifically within the JAK2/STAT3 pathway, which is triggered by IL-6 [60]. The gene expression of IL-6 is also regulated by the canonical AhR pathway. Upon activation by kynurenine, AhR induces IL-6 production, which subsequently activates STAT3. This activation of STAT3 results in the upregulation of IDO expression.

This IDO-AhR-IL-6-STAT3 signaling loop appears to be responsible for the elevated expression of IDO in several human tumors, contributing to immune evasion, a hallmark of the most undifferentiated and aggressive cancers [61]. Clinical studies have linked the activation of the aforementioned circuit with an increased risk of recurrence and reduced survival in patients with lung cancer [62].

In human hepatocellular carcinoma cells, approximately 2 kilobases upstream of the AhR gene’s transcription start site, there are DNA sequences sensitive to STAT3 activity, referred to as STAT-responsive elements. When STAT3 is activated by IL-6, it binds to these specific elements, leading to the activation of AhR gene transcription. The first phase of neoplasia development involves mechanisms and pathways similar to those of inflammation [63]. The IDO-AhR-IL-6-STAT3 loop, activated in response to inflammatory stimuli, may therefore represent a link between the initial phase of carcinogenesis and carcinogenic agents. This is because, in addition to kynurenine, a xenobiotic can also activate this loop.

### 4.2. Indirect Interplay Between STAT3 and AhR

Indirect interplay refers to non-genomic AhR pathways, such as those mediated by Src As previously described, Src is a cytosolic kinase within the latent AhR protein complex. Upon ligand binding by AhR, Src dissociates from the complex and becomes activated. Src then exerts its kinase activity by activating ERK1/2-mediated pathways and/or by phosphorylating receptors like the epidermal growth factor receptor (EGFR). EGFR pathways are essential for regulating cell division and growth, and they are also mediated by the STAT3 protein [64]. In tissues where AhR, Src, EGFR, and STAT3 are co-expressed, the AhR response to environmental pollutants is not merely mediated by the canonical genomic and non-genomic AhR pathways, but also enhanced by the activity of STAT3 [65].

Some studies have demonstrated that the AhR-Src-STAT3 signaling pathway is responsible for the activation of IL-10 gene transcription, a critical pathway in the immune regulation of inflammatory macrophages [66]. Consequently, AhR could serve as a potential therapeutic target for modulating immune responses. The non-genomic interplay between AhR-Src-STAT3, in the presence of persistent and bioaccumulative environmental pollutants, is continuously triggered. As a result, escaping the on–off regulation, it becomes constitutively activated, thereby contributing to the process of carcinogenesis.

Another indirect interaction between AhR and STAT3 involves NADPH oxidase 1 (NOX1), an enzyme responsible for generating reactive oxygen species (ROS). NOX1 is regulated in two ways: through the canonical AhR pathway, which mediates its gene transcription, and through the non-genomic pathway that modulates cytosolic calcium concentration, a known activator of NOX1 [67,68].

In the interplay between AhR, STAT3, and NOX1, another protein involved is pyruvate kinase M2 (PKM2). Pyruvate kinase (PK) is a glycolytic enzyme, but in tumors, the M2 isoform (PKM2) is involved in the onset of the Warburg effect [69]. The literature describes an interaction between this protein and STAT3 in the regulation of tumor metabolic processes [70,71]. Therefore, an additional interplay between AhR-NOX1-ROS-PKM2-STAT3 can be hypothesized, potentially playing a role in the onset and progression of tumors in response to environmental pollutant-induced insults.

Additionally, ROS, particularly through the AhR-NOX1-ROS axis, plays a role in activating non-canonical pathways of STAT3 via its phosphorylation on Serine 727 and mitochondrial localization. This, in turn, influences the activity of the electron transport chain. Since phosphorylation at serine 727 of STAT3 is characteristic of more undifferentiated tumor forms [72], the interplay between the latter and the AhR-NOX1-ROS axis plays a significant role even in the later stages of chemical carcinogenesis.

### 4.3. Potential Therapeutic Target in AhR- STAT3 Interplay

The proteins involved in the direct and indirect interplays between AhR and STAT3, as described and summarized in Table 1, may serve as potential therapeutic targets in cancer treatment. Notably, the IDO and IL-6 protein receptors, which participate in the first direct genomic interplay (IDO-AhR-IL6-STAT3), represent promising candidates. IDO is overexpressed in tumor cells and is already being investigated for inhibitory compounds, some of which have entered Phase 1 clinical trials [73,74].

In contrast, IL-6 exerts its effects through cytokine receptors with tyrosine kinase activity (gp130/JAK2), which could serve as pharmacological targets. Studies on JAK2 inhibitors, some of which are already utilized in cancer therapies, have been reported in the literature [75,76,77]. In the non-genomic indirect interplay (AhR-Src-STAT3), the Src protein has been identified as a promising therapeutic target in gastric cancer. Moreover, the therapeutic efficacy of saracatinib, a specific Src inhibitor, has been evaluated in this context [78,79].

In the AhR-NOX1-ROS-PKM2-STAT3 interplay, NOX1 emerges as a protein of particular interest. NOX1 is implicated in the development of Ras-dependent tumors and represents a potential therapeutic target [80,81]. Within the AhR-STAT3 loops, STAT3 itself is a key target, as it drives the production of reactive oxygen species (ROS), which in turn contribute to PKM2 activation and STAT3 phosphorylation at the Y705 residue. Notably, ROS also play a critical role in altering tumor energy metabolism and activating other oncogenic pathways [82]. This suggests that pharmacological inhibition of NOX1 could be an effective strategy for mitigating chemical carcinogenesis. The study of the interplay between the two key players in chemical carcinogenesis, AhR and STAT3, has led to the identification of four additional biotargets—IDO, IL-6, Src, and NOX1. This advancement enables the development of diverse cancer therapies and also opens the possibility of combination therapy, which may enhance pharmacological efficacy.

## 5. Conclusions

Understanding the cellular and molecular mechanisms responsible for the onset and progression of carcinogenesis induced by xenobiotics is crucial, given the significant impact environmental pollution has on health. As reported by numerous studies, there is a noticeable increase in cancer cases among populations residing in polluted areas. These pollutants are estimated to be responsible for approximately 9 million deaths annually, accounting for 16% of all global deaths. This figure is three times greater than the combined mortality caused by AIDS, tuberculosis, and malaria [83].

In chemical carcinogenesis, key roles are played by pathways mediated by AhR, oncoprotein STAT3, and their interplay. AhR is the primary protein responsible for detoxification and metabolism of xenobiotics, whereas STAT3, as previously described, is crucial in all three stages of carcinogenesis and also functions as an essential hub protein in cellular responses to environmental pollutant insults. Therefore, understanding not only the individual pathways involved in chemical carcinogenesis but also their potential interplay is essential for comprehending the broader process of chemical carcinogenesis and for identifying potential therapeutic approaches and prevention strategies.

Focusing not only on the key proteins involved in a cellular process—AhR and STAT3 in this context—but also on their potential interactions allows for the identification of additional biocomponents. This, in turn, expands the possibility of discovering new targets for a specific process, which can be utilized both as biotargets and biomarkers.

As the renowned Chinese philosopher Sun Tzu stated over 2500 years ago: ’Know yourself and know your enemy to win in war’ [84].

## 6. Future Perspectives

Understanding how environmental factors influence chemical carcinogenesis regulated by AhR-STAT3 loops can aid scientists and clinicians in developing targeted therapies to inhibit carcinogenesis. Furthermore, since there is extensive literature describing the cellular processes controlled by individual components of the loops, it is possible to predict which diseases may develop in the human organism following specific environmental insults. This enables the planning of targeted healthcare prevention and personalized therapy tailored to the specific type of pollutant.

## Figures and Tables

**Table 1:** Schematic overview of the interplays between AhR-STAT3.

Interplays AhR-STAT3		References
Direct Interplay	IDO/AhR/IL-6/STAT3	[61]
Indirect Interplays	AhR/Src/STAT3	[66]
	AhR/NOX1/ROS/STAT3	[67,68]
	AhR/NOX1/ROS/PKM2/STAT3	[69,70,71]
